# A Machine Learning–Derived Risk Scorecard for Pneumonia Hospitalization in Japanese Old‐Old Adults

**DOI:** 10.1111/ggi.70850

**Published:** 2026-09-24

**Authors:** Akio Shimizu, Hajime Kamiya, Masaki Tanabe, Ryota Sakamoto, Hirokazu Matsui, Ryo Momosaki

**Affiliations:** ^1^ Department of Rehabilitation Medicine Mie University Hospital Tsu Japan; ^2^ Department of Public Health, Occupational Medicine, and Applied Epidemiology Mie University Graduate School of Medicine Tsu Japan; ^3^ Department of Infection Control and Infectious Disease Crisis Management Mie University Graduate School of Medicine Tsu Mie Japan; ^4^ Department of Medical Informatics Mie University Hospital Tsu Japan; ^5^ Department of Mechanical Engineering Mie University Graduate School of Engineering Tsu Japan

**Keywords:** frailty, machine learning, pneumonia, QMCOO, risk scorecard

## Abstract

**Aim:**

To develop and internally validate a machine learning–based risk scorecard for 1‐year pneumonia hospitalization among community‐dwelling Japanese adults aged ≥ 75 years using routinely collected frailty screening and claims data.

**Methods:**

We conducted a retrospective cohort study of 1 098 404 community‐dwelling adults aged ≥ 75 years who completed the Questionnaire for Medical Checkup of Old‐Old (QMCOO) between April 2020 and March 2024, using the Late‐Stage Medical Care System claims database. Data were split into training (70%) and test (30%) sets. A Super Learner ensemble of 20 base learners was developed to predict 1‐year pneumonia hospitalization (ICD‐10: J12–J18, J69). An independent 17‐feature point‐based scorecard (0–21 points) was derived from the training set with Platt calibration. Performance was evaluated by the area under the receiver operating characteristic curve (AUC), calibration slope, and calibration‐in‐the‐large.

**Results:**

Among 1 098 404 participants (mean age 80.6 [SD 5.0] years; 40.3% male), 4525 (0.41%) experienced pneumonia hospitalization. On the test set, the Super Learner achieved an AUC of 0.823 (95% CI, 0.812–0.834) and the scorecard 0.786 (0.774–0.798), which showed good calibration (slope 1.017; calibration‐in‐the‐large −0.001). Stratification into low (0–6 points; 55.6%, 0.12% event rate), moderate (7–10; 35.4%, 0.49%), and high (≥ 11; 9.0%, 1.91%) groups yielded a 15.9‐fold risk gradient.

**Conclusions:**

A QMCOO‐based risk scorecard showed good discrimination and calibration for predicting 1‐year pneumonia hospitalization in this population. If externally validated, this tool may help identify high‐risk individuals during routine health checkups and support targeted preventive assessment in primary care.

## Graphical abstract

In 1 098 404 community‐dwelling Japanese adults aged ≥ 75 years, a 20‐learner Super Learner using routinely collected health checkup and claims data predicted 1‐year pneumonia hospitalization (test‐set AUC 0.823). An independently developed, well‐calibrated 17‐feature scorecard showed AUC 0.786 and a 15.9‐fold low‐to‐high risk gradient; external validation is required before clinical use.

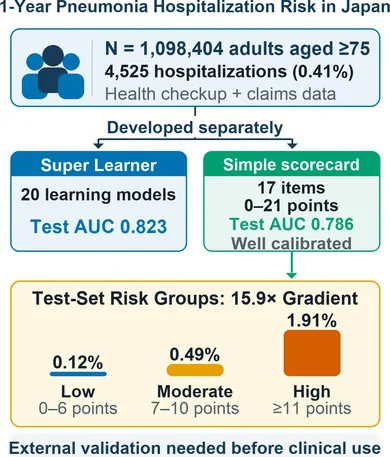

## 1 Introduction

Community‐acquired pneumonia (CAP) is one of the leading causes of hospitalization and death among older adults, with incidence increasing sharply after the age of 75 years [1]. In Japan, the annual incidence of community‐onset pneumonia has been estimated at 1690 per 100 000 population aged 15 years and older, with the incidence in those aged ≥ 85 years approximately 10‐fold that of adults aged 15–64 years [2]. Among older adults hospitalized with CAP, the in‐hospital mortality rate has been reported to range from 2% to 30% [3]. In addition to adverse outcomes in the acute phase, hospitalization for CAP may also result in functional decline [4]. Prolonged bed rest and physical inactivity during hospitalization may contribute to muscle weakness and reduced physical function in older adults [5, 6], and hospitalization itself has also been associated with subsequent cognitive decline [7]. In addition, functional decline during hospitalization for CAP often persists after discharge [8] and is associated with an increased risk of subsequent readmission or mortality [9]. Therefore, prevention of CAP in older adults may help improve prognosis and reduce the burden on health care systems.

Many risk factors for pneumonia hospitalization in older adults are potentially modifiable. Frailty‐related conditions, including impairments in physical, cognitive, oral, and nutritional status, have been recognized as important risk factors for pneumonia [10, 11, 12]. Because these vulnerabilities frequently coexist, early identification of individuals at high risk of pneumonia may support risk stratification and prioritization of preventive interventions. In addition, accurate identification of high‐risk individuals in the community may enable earlier preventive action before hospitalization becomes necessary. These considerations highlight the importance of developing practical approaches to identify older adults at elevated risk of pneumonia hospitalization in community settings.

Routinely collected health data may help identify older adults at high risk of pneumonia. In Japan, the 15‐item Questionnaire for Medical Checkup of Old‐Old (QMCOO) is administered annually as part of the mandatory health checkup program for individuals aged 75 years and older to prevent frailty [13, 14, 15, 16, 17]. The QMCOO has been validated as a comprehensive frailty indicator [13, 18]. Although the QMCOO total score is useful as a general frailty indicator, it weights all items equally and therefore cannot reflect differences in each item's contribution to pneumonia risk. In addition, QMCOO questionnaire items alone do not capture clinical risk factors such as comorbidities and medication use, limiting their utility for disease‐specific prediction. Combining QMCOO items with claims‐based clinical indicators and applying machine learning, which can account for complex correlations among variables, may enable prediction of pneumonia hospitalization. However, to our knowledge, no study has developed such a model using a comprehensive frailty indicator. An interpretable risk scorecard would also be needed for practical implementation by municipalities.

Therefore, the aim of the present study was to develop and internally validate a machine learning model to predict pneumonia hospitalization among community‐dwelling individuals aged 75 years and older using routinely collected QMCOO and claims data, and to develop a clinically interpretable risk scorecard.

## 2 Methods

### 2.1 Study Design and Data Source

This was a retrospective cohort study using the JMDC Old‐Old Medical Claims Database (JMDC Inc., Tokyo, Japan), which contains anonymized claims data from the Late‐Stage Medical Care System for individuals aged 75 years and older across Japan. The database includes QMCOO health checkup data, inpatient and outpatient medical claims, and prescription records. The specific regions and number of municipalities contributing data are not disclosed in accordance with the Act on the Protection of Personal Information.

### 2.2 Ethical Considerations

The database provided by JMDC Inc. used in this study was fully anonymized to prevent identification of individual patients. Accordingly, the Ethics Committee of Mie University Hospital exempted this study from the requirement for ethical review. Because the database was fully anonymized, the requirement for informed consent was waived. This study followed the TRIPOD statement [19] and the TRIPOD + AI extension [20].

### 2.3 Study Population

Individuals aged 75 years and older who completed the QMCOO between April 2020 and March 2024 were identified from the database. For individuals with multiple health checkups during the study period, only the first checkup within the study window was used to ensure between‐individual independence. The eligibility criteria were as follows: (1) complete responses to all 15 QMCOO items; (2) available body mass index (BMI) data; and (3) at least 1 year of follow‐up or occurrence of the study outcome within 1 year. A complete‐case approach was adopted: individuals with missing data on age, sex, or BMI were excluded. In addition, individuals with a history of pneumonia‐related hospitalization (ICD‐10: J12–J18 and J69) or current gastrostomy use were excluded because they were considered likely to have dysphagia or an inherently high risk of pneumonia. The primary models therefore estimate the 365‐day risk of pneumonia hospitalization in the outcome‐ascertainable population (pneumonia observed, or alive and continuously observed without pneumonia through day 365), rather than the marginal cumulative incidence among all otherwise‐eligible screened individuals.

### 2.4 Outcome

The primary outcome was the first hospitalization for pneumonia within 365 days after the QMCOO screening date (index date). Pneumonia hospitalization was identified using ICD‐10 codes J12–J18 and J69 recorded as the primary reason for hospitalization (admission‐triggering diagnosis) in the inpatient claims data; cases in which these codes appeared only as secondary diagnoses were not counted as events, thereby minimizing misclassification of hospital‐acquired pneumonia. This definition aligned with prior Japanese claims‐based research on pneumonia hospitalization in older adults [12]. The validity of respiratory disease diagnoses in Japanese administrative claims data has been confirmed, with high specificity and positive predictive values (PPVs) for pneumonia codes in the Diagnosis Procedure Combination database [21].

### 2.5 Predictors

A total of 20 candidate predictors were selected based on clinical relevance. These included all 15 items from the QMCOO—a condensed version of the 25‐item Kihon Checklist (KCL) mandated for health checkups in the Late‐Stage Medical Care System [13, 22]. The QMCOO items cover 10 categories: health status (self‐rated health), mental health, dietary habits, oral function (chewing, swallowing), weight change, physical function and falls (gait speed, falls, exercise habit), cognitive function (memory loss, time orientation), smoking, social engagement (going out, social relations), and social support. Three demographic variables (age, sex, and BMI) and two clinical variables (Charlson comorbidity index [CCI] [23] score, calculated from ICD‐10 codes in claims within 1 year prior to screening and number of unique prescribed medications within 90 days prior to screening) were also included. All QMCOO items were dichotomized according to standard scoring criteria. Of the 15 items, 12 are considered frailty‐related and have been shown to form a five‐domain structure [13, 22]. The QMCOO has shown comparable effectiveness to the full 25‐item KCL in this population [15], and the KCL‐based frailty screening has been validated against phenotype‐based frailty criteria [24]. The individual QMCOO items are shown in Table S1.

### 2.6 Statistical Analysis

Individuals were randomly allocated to training (70%) and test (30%) sets, stratified by outcome and Japanese fiscal year, with no individual appearing in both sets. All feature selection, model tuning, and probability calibration used the training set only, and the test set was reserved for a single final evaluation. A Super Learner ensemble of 20 base learners (generalized linear and additive models, penalized regression, gradient boosting machines [XGBoost], random forests, multivariate adaptive regression splines, and neural networks) was combined by non‐negative least squares with 10‐fold outcome‐stratified cross‐validation [25]. Given the low prevalence (0.41%), we deliberately did not apply class‐rebalancing (random over‐ or under‐sampling, class weighting, or synthetic minority over‐sampling). The natural prevalence was retained, cross‐validation folds were stratified by outcome, and predicted probabilities were calibrated by Platt scaling, because artificial rebalancing can inflate baseline risk and impair the calibration of clinical risk‐prediction models [26]. Variable importance was assessed by permutation (decrease in the area under the receiver operating characteristic curve [AUC] on permuting each variable).

An interpretable scorecard was developed independently of the Super Learner by pruning correlated features, ranking the remainder using least absolute shrinkage and selection operator (LASSO), ridge regression, and analysis of variance, selecting the number of features by internal cross‐validation, and fitting a Platt‐calibrated logistic model. Information value summarized each selected feature's predictive strength. Binary features scored 0–1 and continuous features 0–2 using training‐set quartile cutpoints adjusted for ties and applied unchanged to the test set. Points were summed, and three descriptive risk groups were defined by training‐set cutpoints yielding monotonically increasing event rates; these were not prespecified clinical thresholds. For calibration and decision curve analysis, the integer total score was separately mapped to a predicted probability by Platt scaling fitted in the training set.

Performance was evaluated by AUC, calibration slope, calibration‐in‐the‐large (CITL), Brier score, and the Kolmogorov–Smirnov statistic, with emphasis on calibration for clinical safety [27]. Clinical utility was assessed by decision curve analysis and, for scores ≥ 7 and ≥ 11, the proportion flagged, sensitivity, specificity, PPV, and number needed to screen (NNS). Prespecified subgroups were sex and age group (75–79, 80–84, 85–89, and ≥ 90 years), with calibration additionally examined in the oldest‐old.

Sensitivity analyses evaluated event rates, discrimination, and calibration by fiscal year (FY2020 [April 2020–March 2021] to FY2023 [April 2023–March 2024]) and competing mortality in the full otherwise‐eligible cohort before exclusion for incomplete 1‐year follow‐up. The Aalen–Johansen 1‐year cumulative incidence of pneumonia, treating non‐pneumonia death as a competing event, was compared with the complement of the Kaplan–Meier estimate, which treated competing death as censoring; 95% CIs were calculated for both estimates. Because death was recorded monthly, robustness was assessed by assigning death to the beginning, middle (Day 15), or end of the month, with month‐end assignment primary. In an additional sensitivity analysis, the final models were evaluated without refitting in the full eligible cohort, treating death before pneumonia as a known non‐event and weighting non‐death loss of observation by inverse‐probability‐of‐censoring weighting (IPCW), with included and excluded individuals compared by standardized mean differences (Supporting Information Methods). All analyses used R version 4.4.1 (R Foundation for Statistical Computing, Vienna, Austria).

## 3 Results

**FIGURE 1:**
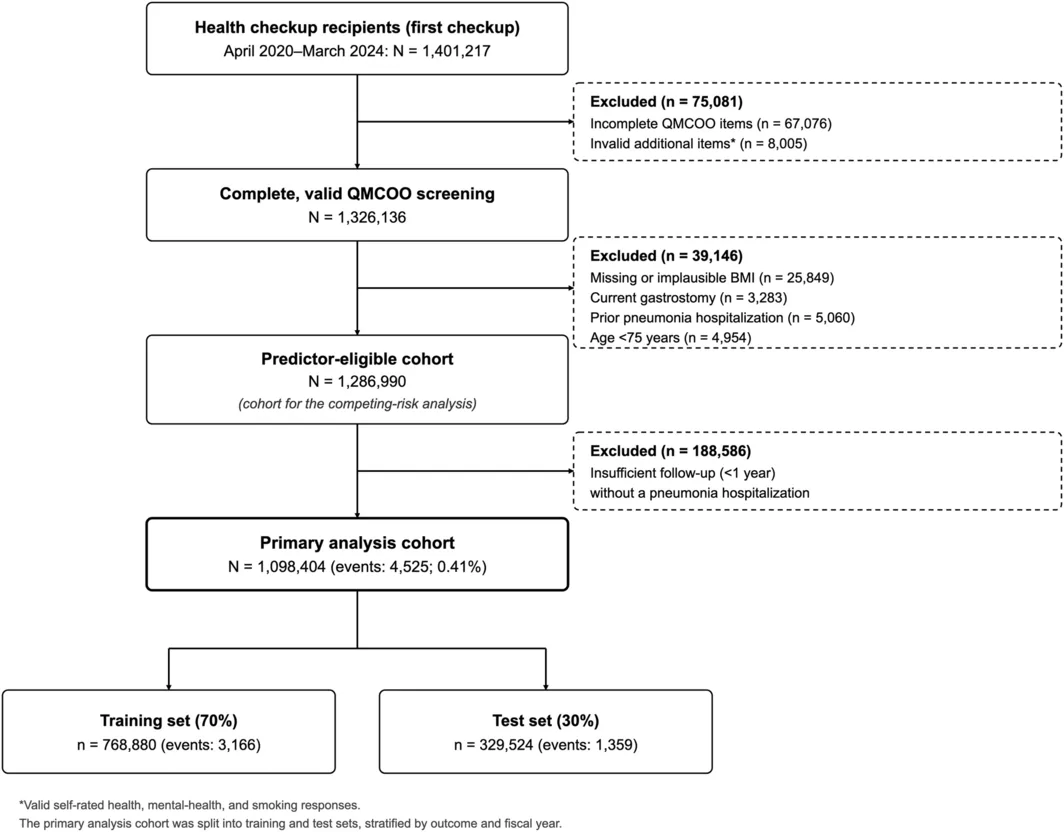
Study flow diagram showing the selection of the study population from the Late‐Stage Medical Care System claims database.

A total of 1 098 404 individuals met the inclusion criteria (Figure 1). Of these, 4525 (0.41%) were hospitalized for pneumonia within 1 year (3166/768 880 in training and 1359/329 524 in test). The mean (standard deviation [SD]) age was 80.6 (5.0) years, 40.3% were male, and mean (SD) BMI was 22.8 (3.4) kg/m^2^ (Table 1). Those hospitalized for pneumonia were older and more often male, had lower BMI, and reported more adverse responses across all 15 QMCOO items. Among participants without the primary outcome, 119 378 (10.9%) had another all‐cause hospitalization.

**TABLE 1:** Baseline characteristics of the study participants according to pneumonia hospitalization status.

Characteristics	Overall (*N* = 1 098 404)	Non‐event (*N* = 1 093 879)	Event (*N* = 4525)
Demographics			
Age, years, mean (SD)	80.6 (5.0)	80.5 (5.0)	84.5 (6.1)
Male sex, *n* (%)	442 691 (40.3)	439 787 (40.2)	2904 (64.2)
BMI (kg/m^2^), mean (SD)	22.8 (3.4)	22.8 (3.4)	21.6 (3.8)
Clinical indicators			
CCI score, mean (SD)	1.7 (1.9)	1.7 (1.9)	2.9 (2.3)
No. of medications, mean (SD)	6.8 (5.5)	6.8 (5.5)	9.3 (6.4)
QMCOO items			
QMCOO total score, mean (SD)	2.7 (2.1)	2.7 (2.1)	4.5 (2.6)
Self‐rated health (poor), *n* (%)	114 406 (10.4)	113 416 (10.4)	990 (21.9)
Mental health (dissatisfied), *n* (%)	107 835 (9.8)	107 045 (9.8)	790 (17.5)
Dietary habit (eat less), *n* (%)	69 568 (6.3)	69 089 (6.3)	479 (10.6)
Chewing difficulty, *n* (%)	299 622 (27.3)	297 663 (27.2)	1959 (43.3)
Swallowing difficulty, *n* (%)	227 249 (20.7)	225 777 (20.6)	1472 (32.5)
Weight loss, *n* (%)	132 030 (12.0)	131 042 (12.0)	988 (21.8)
Slow walking speed, *n* (%)	661 776 (60.2)	658 272 (60.2)	3504 (77.4)
Falls in the past year, *n* (%)	195 362 (17.8)	193 876 (17.7)	1486 (32.8)
Lack of exercise habit, *n* (%)	398 173 (36.3)	395 659 (36.2)	2514 (55.6)
Memory complaints, *n* (%)	176 304 (16.1)	174 697 (16.0)	1607 (35.5)
Difficulty knowing date, *n* (%)	272 709 (24.8)	270 676 (24.7)	2033 (44.9)
Current smoker, *n* (%)	57 980 (5.3)	57 730 (5.3)	250 (5.5)
Reduced going out, *n* (%)	110 161 (10.0)	109 000 (10.0)	1161 (25.7)
Limited social relations, *n* (%)	68 004 (6.2)	67 360 (6.2)	644 (14.2)
Lack of social support, *n* (%)	58 199 (5.3)	57 922 (5.3)	277 (6.1)

*Note:* Values are presented as mean (SD) or *n* (%).

Abbreviations: BMI, body mass index; CCI, Charlson comorbidity index; QMCOO, Questionnaire for Medical Checkup of Old‐Old; SD, standard deviation.

The Super Learner achieved a 10‐fold cross‐validated AUC of 0.815 (95% CI, 0.807–0.822) and a test‐set AUC of 0.823 (0.812–0.834) (Table 2; Figure S1). On the test set, the Brier score was 0.0041, calibration slope 1.101, and CITL −0.002 (Figure S2). Male sex, BMI, age, medication count, and comorbidity burden contributed most by permutation importance (Table S2).

**TABLE 2:** Performance of the Super Learner ensemble and risk scorecard on the test set.

Metric	Super Learner	Risk scorecard (integer)
AUC (95% CI)	0.823 (0.812–0.834)	0.786 (0.774–0.798)
Brier score	0.0041	0.0041
Calibration slope	1.101	1.017
Calibration‐in‐the‐large	−0.002	−0.001

*Note:* All metrics are from the test set (30% holdout).

Abbreviations: AUC, area under the receiver operating characteristic curve; CI, confidence interval; CITL, calibration‐in‐the‐large.

A parallel 17‐feature integer score (range 0–21) was derived from the training set; ridge‐ranked internal cross‐validation selected 17 features after correlation filtering (Figure S3). Its test‐set AUC was 0.786 (95% CI, 0.774–0.798), with calibration slope 1.017, CITL −0.001, and Brier score 0.0041 (Table 2; Figures S1 and S2). The Super Learner AUC exceeded the integer‐score AUC by 0.037 (95% CI, 0.030–0.044). The scoring scheme and feature information values are shown in Tables 3 and S3.

**TABLE 3:** Risk scorecard for pneumonia hospitalization prediction.

Feature	Category	Score	Event rate (%)	*N*
Age, years	≤ 76	0	0.18	206 559
	77–84	1	0.31	395 733
	≥ 85	2	0.94	166 588
Male sex	No	0	0.25	459 436
	Yes	1	0.65	309 444
CCI score	≤ 1	0	0.24	429 562
	2–3	1	0.50	221 502
	≥ 4	2	0.87	117 816
BMI, kg/m²	≤ 20.5	2	0.69	192 314
	20.6–24.9	1	0.33	389 444
	> 24.9	0	0.30	187 122
No. of medications	≤ 3	0	0.24	232 996
	4–9	1	0.37	344 617
	≥ 10	2	0.71	191 267
Self‐rated health	Good	0	0.36	688 911
	Poor	1	0.86	79 969
Chewing difficulty	No	0	0.32	559 562
	Yes	1	0.66	209 318
Swallowing difficulty	No	0	0.35	609 869
	Yes	1	0.64	159 011
Slow walking speed	No	0	0.23	305 593
	Yes	1	0.53	463 287
Falls	No	0	0.34	632 285
	Yes	1	0.75	136 595
Lack of exercise habit	No	0	0.28	490 236
	Yes	1	0.64	278 644
Memory complaints	No	0	0.32	645 335
	Yes	1	0.91	123 545
Difficulty knowing date	No	0	0.30	577 943
	Yes	1	0.75	190 937
Reduced going out	No	0	0.34	692 081
	Yes	1	1.05	76 799
Weight loss	No	0	0.37	676 509
	Yes	1	0.74	92 371
Limited social relations	No	0	0.38	721 240
	Yes	1	0.93	47 640
Dietary habit (eat less)	No	0	0.39	720 273
	Yes	1	0.71	48 607

*Note:* Total score range: 0–21 (13 binary items × 0–1, 4 continuous variables × 0–2). Risk groups: low (0–6), moderate (7–10), and high (≥ 11). Event rates are from the training set. Adjacent quartiles that received equal points are shown as a single band.

Abbreviations: BMI, body mass index; CCI, Charlson comorbidity index.

**FIGURE 2:**
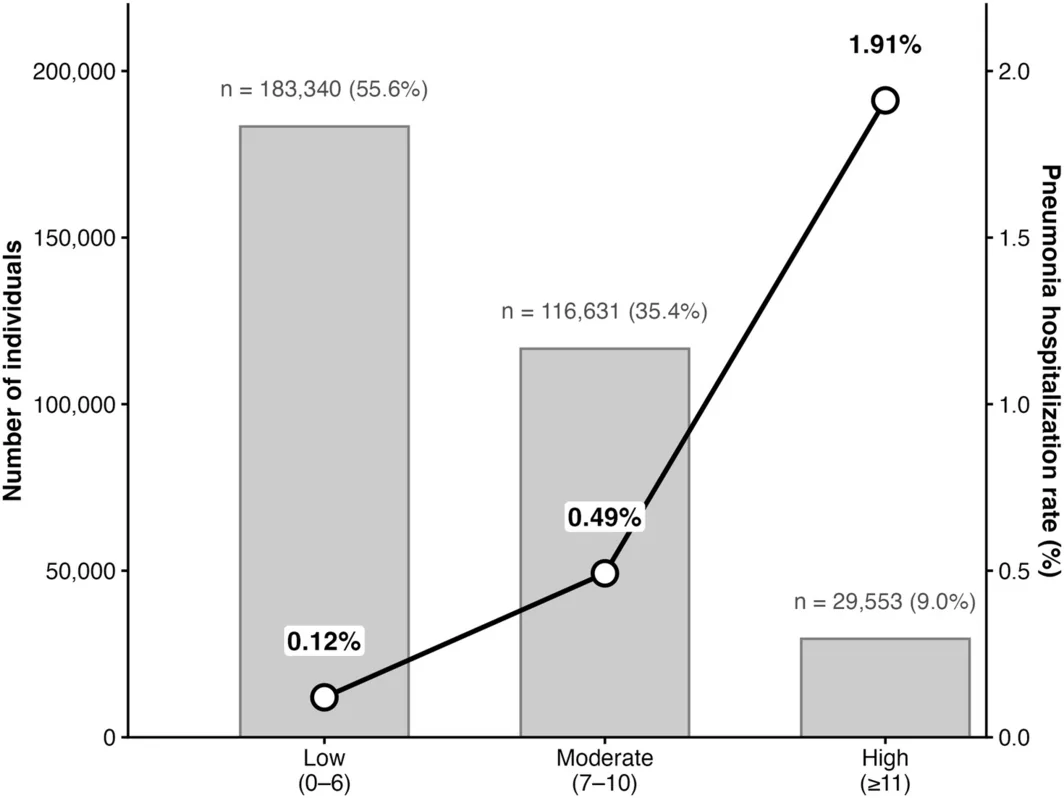
Risk stratification by the 17‐feature risk scorecard in the test set. Bars represent the number of individuals in each risk group (left *y*‐axis), and the line with markers indicates the observed 1‐year pneumonia hospitalization rate (right *y*‐axis). The event rate was 15.9‐fold higher in the high‐risk group (1.91%) than in the low‐risk group (0.12%).

Training‐set‐derived cutoffs defined low (0–6 points; *n* = 183 340 [55.6%]; event rate 0.12%), moderate (7–10; *n* = 116 631 [35.4%]; 0.49%), and high (≥ 11; *n* = 29 553 [9.0%]; 1.91%) groups in the test set, a 15.9‐fold gradient (Kolmogorov–Smirnov statistic 0.44) (Figure 2). A score ≥ 7 flagged 44.4% of participants and captured 83.8% of events (PPV 0.78%; NNS 128), whereas a score ≥ 11 flagged 9.0% and captured 41.6% (PPV 1.91%; NNS 52) (Table S4). Decision curve analysis showed positive net benefit across clinically relevant threshold probabilities (Figure S4).

Integer‐score AUC was 0.776 in both women and men, and 0.752, 0.743, 0.723, and 0.670 for ages 75–79, 80–84, 85–89, and ≥ 90 years, respectively (Table S5). Among participants aged ≥ 90 years (*n* = 19 725; 299 events; event rate 1.52%), the AUC was 0.670 (95% CI, 0.639–0.700); scores were higher and more narrowly distributed than in younger participants (median 9 [interquartile range 7–11] vs. 6 [4, 5, 6, 7, 8]), with a calibration slope of 0.452 and CITL +0.586.

Full‐cohort event rates were 0.37%, 0.39%, 0.66%, and 0.39% in FY2020–FY2023, and corresponding test‐set integer‐score AUCs were 0.803, 0.787, 0.810, and 0.779; risk underestimation was greatest in FY2022 (CITL +0.796; Table S6). In a competing‐risk analysis of 1 286 990 otherwise‐eligible participants, there were 4525 pneumonia events and 18 138 non‐pneumonia deaths within 365 days (Figure 1). The Aalen–Johansen pneumonia incidence was 0.378% (95% CI, 0.367–0.389), differing by 0.003 percentage points from the complement of the Kaplan–Meier estimate (0.381%; 0.370–0.392). Estimates ranged from 0.354% to 0.378% across death‐timing assignments (Table S7; Figure S5).

In the full‐eligible‐cohort evaluation, excluded decedents were markedly frailer than included individuals (Table S8). Discrimination of the final models was largely preserved (IPCW‐weighted AUC 0.818 [95% CI, 0.806–0.829] and 0.782 [0.768–0.794]; changes −0.005 and −0.003), predictions modestly overestimated population risk (integer‐score slope 0.971; CITL −0.080; predicted‐to‐observed ratio 1.10), and all 17 refitted coefficients agreed in direction (Tables S9–S11); the integer score underestimated risk at ages ≥ 90 years (CITL +0.31).

## 4 Discussion

This study showed that 1‐year pneumonia hospitalization risk among community‐dwelling old‐old adults can be predicted with good discrimination using routinely collected health checkup and claims data. The Super Learner ensemble showed good discrimination, and a parallel sum‐based risk scorecard retained most of the ensemble's discrimination with good calibration. This is the first study to develop, in a large population of old‐old adults, a pneumonia risk model that pairs a Super Learner with an interpretable scorecard.

An important contribution of this study is that we independently developed a simple, interpretable scorecard alongside the Super Learner. The Super Learner is difficult to interpret, and its flexible learners may overfit a rare outcome. The scorecard mitigates these concerns. This simple model requires no specialized software. However, the scorecard had a lower AUC than the Super Learner (0.786 vs. 0.823). Li et al. [25] similarly reported a small decrease in AUC when predicting metabolic syndrome. In terms of calibration, the scorecard's predicted risks more closely matched the observed event rates than the Super Learner's predictions, as shown by a calibration slope closer to 1 (1.017 vs. 1.101) and a CITL closer to 0 (−0.001 vs. −0.002). Beyond these performance measures, its key predictors—male sex, older age, low BMI, and comorbidity burden—are consistent with known pneumonia risk factors [1, 28], making the model easier to interpret clinically.

Selected predictors may contribute through different pathways. Reduced physical activity may impair cough through sarcopenia and respiratory‐muscle weakness [29]; chewing and swallowing difficulty may increase aspiration risk and impair airway defense [10, 12]; and cognitive decline and social isolation may act through poor oral hygiene, nutrition, and delayed care‐seeking [30]. However, these observational predictors are prognostic markers rather than causal or modifiable intervention targets; medication count may reflect comorbidity and health care utilization rather than biological risk. These pathways are hypotheses that cannot be confirmed from the present observational design.

The scorecard can be implemented within Japan's Late‐Stage Medical Care System without additional data collection because the QMCOO is already used in health screening for adults aged ≥ 75 years [13] and is validated for frailty assessment [15, 31]. Risk stratification may help target oral health care [32], pneumococcal vaccination, swallowing and nutritional assessment, and exercise. Although the event rate was low (0.41%), community‐onset pneumonia incidence is substantially higher [2], and pneumonia remains a leading cause of death in older adults [1]. Whether scorecard‐based identification improves outcomes warrants prospective validation. The complete scorecard specification, including a lookup table mapping each integer score (0–21) to the calibrated 1‐year probability, is provided in Tables S12 and S13.

These data‐driven risk groups illustrate a precision–coverage trade‐off. The cutpoints were selected to yield monotonically increasing event rates. A score ≥ 11 provides a narrower target for intensive individualized assessment, whereas a score ≥ 7 provides a broader target for scalable, low‐intensity prevention. Neither threshold is endorsed for clinical use without external validation. The optimal operating point depends on intervention effectiveness, harms, costs, and available resources.

Lower discrimination among adults aged ≥ 90 years may reflect their higher, narrower score distribution. Frailty and comorbidity may also be less pneumonia‐specific at very advanced ages; greater competing mortality may attenuate associations, and determinants may shift toward factors not captured here. Age‐specific recalibration and measures of swallowing, nutritional status, functional dependency, and long‐term‐care need may improve prediction.

This study has several limitations. Health checkup attendees may be healthier than non‐attendees, limiting generalizability to frailer or homebound populations. QMCOO items were self‐reported, with possible recall bias, and vaccination status, oral hygiene, and nutritional intake were unavailable, leaving residual confounding. The low event rate limited the PPV; although the low‐to‐high gradient was 15.9‐fold, 9.0% were high risk, and the small positive scaled Brier scores (1.3% ensemble; 0.7% integer score) should be considered alongside discrimination and calibration. The lower PPV of ICD‐10 code J69 than J12–J18 may have caused outcome misclassification [21]. Because development required a determinable 1‐year outcome, the primary estimand is conditional on outcome ascertainment; the full‐cohort evaluation showed preserved discrimination but modest overestimation of population risk, so recalibration would be advisable before deployment targeting all screened individuals, and residual informative disenrollment cannot be excluded.

Non‐pneumonia death was a substantial competing event (18 138 deaths; cumulative incidence 1.53% vs. 0.38% for pneumonia), yet accounting for it changed estimated pneumonia incidence only marginally (0.378% vs. 0.381%; 0.354%–0.378% across death‐timing assumptions). Pandemic‐era event rates fluctuated (0.37%–0.66%); discrimination was broadly stable, but calibration shifted, so temporal recalibration may be needed. Finally, external validation is essential [33], particularly in metropolitan and rural municipalities differing in population aging, hospital‐bed density, and access to care, and in post‐pandemic cohorts, with prospective recalibration and clinical‐impact assessment.

## 5 Conclusions

A QMCOO‐based risk scorecard showed good discrimination and calibration for predicting 1‐year pneumonia hospitalization among community‐dwelling adults aged ≥ 75 years. If externally validated, it may help identify high‐risk individuals during routine health checkups and guide targeted preventive assessment in primary care. Prospective validation and assessment of clinical impact are needed before widespread implementation, and independent validation with age‐specific recalibration remains necessary before clinical use in people aged ≥ 90 years.

## Author Contributions

**Akio Shimizu**, **Hajime Kamiya**, and **Ryo Momosaki:** conceptualization. **Akio Shimizu** and **Ryota Sakamoto:** data curation. **Akio Shimizu** and **Hirokazu Matsui:** formal analysis and software. **Akio Shimizu:** funding acquisition, visualization, and writing – original draft. **Akio Shimizu**, **Hajime Kamiya**, and **Masaki Tanabe:** investigation. **Akio Shimizu**, **Hajime Kamiya**, Masaki Tanabe, and **Hirokazu Matsui: methodology**. **Akio Shimizu** and **Ryo Momosaki:** project administration and resources. **Hajime Kamiya** and **Ryo Momosaki:** supervision. **Akio Shimizu**, **Hajime Kamiya**, **Masaki Tanabe**, and **Ryota Sakamoto:** validation. **Akio Shimizu**, **Hajime Kamiya**, **Masaki Tanabe**, **Ryota Sakamoto**, **Hirokazu Matsui**, and **Ryo Momosaki:** writing – review and editing. All authors have read and approved the final version of the manuscript and agree to be accountable for all aspects of the work.

## Funding

This work was supported by the Okasan Kato Culture Promotion Foundation (Grant Number 2025‐1‐30) and JSPS KAKENHI (Grant Number JP25K02851). The funders had no role in study design, data collection, analysis, interpretation, manuscript preparation, or the decision to submit the manuscript for publication.

## Ethics Statement

The Ethics Committee of Mie University Hospital exempted this study from the requirement for ethical review because the database used was fully anonymized and did not allow identification of individual patients.

## Consent

Informed consent was waived because the database was fully anonymized in accordance with the Act on the Protection of Personal Information.

## Conflicts of Interest

The authors declare no conflicts of interest.

## Supporting information


**Table S1:** The 15‐item Questionnaire for Medical Checkup of Old‐Old.
**Table S2:** Permutation variable importance of the Super Learner model (top 15 predictors).
**Table S3:** Information value and logistic regression β coefficients of the 17 selected features in the risk scorecard.
**Table S4:** Decision‐analytic screening metrics for alternative intervention targets (test set).
**Table S5:** Discriminative performance of the integer risk scorecard by subgroup (sex and age group) in the test set.
**Table S6:** Time‐stratified (fiscal‐year) sensitivity analysis of the integer risk scorecard during the COVID‐19 pandemic period.
**Table S7:** Competing‐risk cumulative‐incidence analysis of pneumonia hospitalization with non‐pneumonia death as a competing event.
**Table S8:** Characteristics of included versus excluded individuals in the full otherwise‐eligible cohort.
**Table S9:** Full‐eligible‐cohort sensitivity evaluation of the final models with death as a competing event and inverse probability of censoring weighting.
**Table S10:** Stability of the 17 scorecard logistic regression coefficients when refitted with IPCW weights in the full‐cohort development analog.
**Table S11:** Decile‐level calibration of the final models in the full‐eligible‐cohort evaluation.
**Table S12:** Complete point‐assignment specification of the 17‐feature integer risk scorecard.
**Table S13:** Lookup table mapping each integer score (0–21) to the calibrated 1‐year predicted probability of pneumonia hospitalization.
**Figure S1:** ROC curves of (a) the Super Learner ensemble and (b) the 17‐feature integer risk scorecard in the test set.
**Figure S2:** Calibration plots of (a) the Super Learner ensemble and (b) the 17‐feature integer risk scorecard in the test set.
**Figure S3:** Incremental feature selection by development‐internal 10‐fold cross‐validation for the three ranking methods (LASSO, ridge, and ANOVA).
**Figure S4:** Decision curve analysis comparing the net benefit of the 17‐feature integer risk scorecard against treat‐all and treat‐none strategies across a range of threshold probabilities in the test set.
**Figure S5:** Competing‐risk cumulative incidence of pneumonia hospitalization and non‐pneumonia (competing) death over 1 year, with 95% confidence intervals.

## Data Availability

The data underlying this study were obtained from JMDC Inc. under a licensing agreement and are not publicly available. Restrictions apply to the availability of these data. Researchers interested in accessing the JMDC Old‐Old Medical Claims Database may contact JMDC Inc. directly for licensing information.
