# Supplementary material for: A Machine Learning–Derived Risk Scorecard for Pneumonia Hospitalization in Japanese Old‐Old Adults
Source: Geriatr Gerontol Int. 2026 Sep 24;26(10):e70850. doi: 10.1111/ggi.70850 (PMC13602450; doi:10.1111/ggi.70850)
Supplement: Supplementary file 1 [file GGI-26-0-s001.docx]

# Supplementary Materials

*A Machine Learning–Derived Risk Scorecard for Pneumonia Hospitalization in Japanese Old-Old Adults*

## Supplementary Methods. Full-eligible-cohort sensitivity analysis with death as a competing event and inverse probability of censoring weighting (IPCW).

The final models were evaluated in the full eligible cohort (N = 1,286,990); the predictive models and point assignments were not refitted, and the only newly fitted quantity was a development-only logistic calibration mapping the integer total score to a probability (Supplementary Table 13). The analysis targeted the 1-year cumulative incidence of pneumonia hospitalization with death as a competing event. The evaluation set (n = 386,099) combined the held-out test set (n = 329,524) with a random 30% sample (n = 56,575) of the 188,586 excluded individuals, stratified by fiscal year and death versus other loss of observation, so that no individual used for model fitting contributed. Death before pneumonia was treated as a known non-event, because a recorded death makes the 365-day outcome determinate; non-death loss of observation before day 365 was treated as censoring and handled by IPCW. The censoring model was a Cox proportional hazards model for non-death loss, conditional on all 20 candidate predictors, fiscal year, and the final scorecard’s linear predictor; weights 1/G(t−) were evaluated at the pneumonia, death, or horizon (day 365) time and truncated at their 1st/99th percentiles (untruncated weights gave nearly identical results). Weight diagnostics were as follows: median 1.00, 95th percentile 2.42, and 99th percentile 3.40; before truncation, the maximum weight was 14.5 and the effective sample size 279,505 of 334,952 outcome-ascertainable individuals; after truncation, the maximum was 3.40 and the effective sample size 288,320; the minimum censoring-survival probability was 0.069, and approximately 1% of weights were capped. A global proportional-hazards test for the censoring model was statistically significant (P < 0.001), as expected at this sample size; the close agreement between truncated and untruncated results and across the three death-timing assignments suggests limited sensitivity to the censoring-model specification. Individuals who died and disenrolled in the same month were classified as deaths using the insurer death flag.

We report the IPCW-weighted area under the receiver operating characteristic curve (AUC; competing deaths retained among controls), IPCW-weighted Brier score, calibration slope and calibration-in-the-large (CITL; positive values indicate observed risk exceeding predicted risk on the logit scale), and the ratio of mean predicted to IPCW-estimated observed risk (E/O), together with decile-level calibration against both the Aalen–Johansen cumulative incidence and the IPCW-weighted event rate (Supplementary Table 11). Confidence intervals are bootstrap percentile intervals (B = 500) re-estimating the censoring model in each replicate, conditional on the final models (including the development-fitted score-to-probability mapping). Results were additionally computed under the three within-month death-timing assignments (month start, day 15, month end [primary]). To assess the stability of predictor associations, the 17-feature logistic model was refitted with IPCW weights in the full-cohort development analogue (n = 900,891; the development set, identical to the 70% training set, plus the 70% of excluded individuals not sampled into the evaluation set), with bootstrap standard errors (B = 200). Included and excluded individuals were compared using standardized mean differences (SMDs), with excluded individuals subdivided into deaths before pneumonia and other losses of observation. All analyses used R version 4.4.1 with the survival package.

## Supplementary Table 1. The 15-item Questionnaire for Medical Checkup of Old-Old (QMCOO).

| Item | Domain | Question | Response options |
| --- | --- | --- | --- |
| Q1 | Health status | How is your health condition? | Excellent / Good / Fair / Poor / Very poor |
| Q2 | Mental health | Are you satisfied with your daily life? | Satisfied / Moderately satisfied / Moderately dissatisfied / Dissatisfied |
| Q3 | Dietary habits† | Do you eat three times a day? | Yes / No |
| Q4 | Oral function† | Do you have any difficulties eating tough foods compared to 6 months ago? | Yes / No |
| Q5 | Oral function† | Have you choked on your tea or soup recently? | Yes / No |
| Q6 | Weight change† | Have you lost 2 kg or more in the past 6 months? | Yes / No |
| Q7 | Physical function† | Do you think you walk slower than before? | Yes / No |
| Q8 | Physical function† | Have you experienced a fall in the past year? | Yes / No |
| Q9 | Physical function† | Do you go for a walk for your health at least once a week? | Yes / No |
| Q10 | Cognitive function† | Do your family or friends point out your memory loss? (e.g., “You ask the same question over and over again.”) | Yes / No |
| Q11 | Cognitive function† | Do you find yourself not knowing today’s date? | Yes / No |
| Q12 | Smoking | Do you smoke? | Yes / No / I quit |
| Q13 | Social participation† | Do you go out at least once a week? | Yes / No |
| Q14 | Social participation† | Do you keep regular communication with your family and friends? | Yes / No |
| Q15 | Social support† | When you are not feeling well, do you have anyone you can talk with? | Yes / No |

†Frailty-related item. The 12 frailty-related items (Q3–Q11, Q13–Q15) form a five-domain structure: motor function (Q7–Q9), nutrition (Q3, Q6), oral health (Q4, Q5), cognitive function (Q10, Q11), and social aspects (Q13–Q15).

## Supplementary Table 2. Permutation variable importance of the Super Learner model (top 15 predictors).

| Rank | Variable | QMCOO domain | AUC decrease |
| --- | --- | --- | --- |
| 1 | Male sex | — | 0.074 |
| 2 | BMI | — | 0.065 |
| 3 | Age | — | 0.062 |
| 4 | No. of medications | — | 0.043 |
| 5 | CCI score | — | 0.040 |
| 6 | Exercise habit | Physical function | 0.021 |
| 7 | Chewing difficulty | Oral function | 0.019 |
| 8 | Difficulty knowing date | Cognitive function | 0.018 |
| 9 | Slow walking speed | Physical function | 0.017 |
| 10 | Falls | Physical function | 0.015 |
| 11 | Memory complaints | Cognitive function | 0.014 |
| 12 | Swallowing difficulty | Oral function | 0.013 |
| 13 | Weight loss | Weight change | 0.011 |
| 14 | Reduced going out | Social participation | 0.011 |
| 15 | Self-rated health | Health status | 0.009 |

Variable importance was calculated as the decrease in training-set AUC when each variable was randomly permuted.

**Abbreviations:** AUC, area under the receiver operating characteristic curve; BMI, body mass index; CCI, Charlson Comorbidity Index; QMCOO, Questionnaire for Medical Checkup of Old-Old.

## Supplementary Table 3. Information value and logistic regression β coefficients of the 17 selected features in the risk scorecard.

| Rank | Feature | Information value | Strength | β coefficient |
| --- | --- | --- | --- | --- |
| 1 | Age | 0.415 | Strong | 0.081 |
| 2 | CCI score | 0.265 | Moderate | 0.113 |
| 3 | Male sex | 0.225 | Moderate | 1.183 |
| 4 | Memory complaints | 0.210 | Moderate | 0.370 |
| 5 | Difficulty knowing date | 0.189 | Moderate | 0.272 |
| 6 | No. of medications | 0.181 | Moderate | 0.032 |
| 7 | Reduced going out | 0.177 | Moderate | 0.376 |
| 8 | Lack of exercise habit | 0.165 | Moderate | 0.456 |
| 9 | Slow walking speed | 0.141 | Moderate | 0.155 |
| 10 | BMI | 0.137 | Moderate | −0.126 |
| 11 | Falls | 0.118 | Moderate | 0.248 |
| 12 | Chewing difficulty | 0.118 | Moderate | 0.202 |
| 13 | Self-rated health | 0.099 | Weak | 0.135 |
| 14 | Limited social relations | 0.070 | Weak | 0.109 |
| 15 | Weight loss | 0.070 | Weak | 0.155 |
| 16 | Swallowing difficulty | 0.067 | Weak | 0.158 |
| 17 | Dietary habits | 0.027 | Weak | 0.121 |

Features are ranked by IV, classified as strong (>0.3), moderate (0.1–0.3), or weak (0.02–0.1). β coefficients are from the logistic regression model fitted on the selected raw features (Platt-calibrated).

**Abbreviations:** BMI, body mass index; CCI, Charlson Comorbidity Index; IV, information value.

## Supplementary Table 4. Decision-analytic screening metrics for alternative intervention targets (test set).

| Intervention target | N flagged (%) | Events captured | Sensitivity | Specificity | PPV (%) | NNS |
| --- | --- | --- | --- | --- | --- | --- |
| High-risk only (score ≥11) | 29,553 (9.0%) | 565 | 0.416 | 0.912 | 1.91 | 52 |
| Moderate + high risk (score ≥7) | 146,184 (44.4%) | 1,139 | 0.838 | 0.558 | 0.78 | 128 |

Metrics are calculated on the held-out test set (N = 329,524; 1,359 events). Sensitivity denotes the proportion of all 1-year pneumonia hospitalizations occurring in the flagged group; the NNS is the number of individuals flagged per event captured.

Restricting intervention to the high-risk group captures 41.6% of future pneumonia hospitalizations while flagging 9.0% of the population, whereas broadening the target to the combined moderate-plus-high-risk group captures the large majority (83.8%) of events at a number needed to screen of 128, informing the trade-off between precision and population coverage.

**Abbreviations:** NNS, number needed to screen; PPV, positive predictive value.

## Supplementary Table 5. Discriminative performance (AUC) of the integer risk scorecard by subgroup (sex and age group) in the test set.

| Subgroup | N | Events | Event rate | AUC (95% CI) |
| --- | --- | --- | --- | --- |
| Overall | 329,524 | 1,359 | 0.41% | 0.786 (0.774–0.798) |
| Sex |  |  |  |  |
| Female | 196,277 | 470 | 0.24% | 0.776 (0.754–0.797) |
| Male | 133,247 | 889 | 0.67% | 0.776 (0.761–0.791) |
| Age group |  |  |  |  |
| 75–79 years | 166,646 | 322 | 0.19% | 0.752 (0.724–0.780) |
| 80–84 years | 91,321 | 342 | 0.37% | 0.743 (0.717–0.768) |
| 85–89 years | 51,832 | 396 | 0.76% | 0.723 (0.699–0.748) |
| ≥90 years | 19,725 | 299 | 1.52% | 0.670 (0.639–0.700) |

**Abbreviations:** AUC, area under the receiver operating characteristic curve; CI, confidence interval.

## Supplementary Table 6. Time-stratified (fiscal-year) sensitivity analysis of the integer risk scorecard during the COVID-19 pandemic period.

| Fiscal year | Cohort N | Cohort events | Event rate (%) | Test-set AUC (95% CI) | Calibration slope | CITL |
| --- | --- | --- | --- | --- | --- | --- |
| FY2020 (Apr 2020–Mar 2021) | 165,529 | 613 | 0.37 | 0.803 (0.772–0.833) | 1.105 | −0.009 |
| FY2021 (Apr 2021–Mar 2022) | 756,910 | 2,987 | 0.39 | 0.787 (0.772–0.801) | 1.026 | −0.132 |
| FY2022 (Apr 2022–Mar 2023) | 88,522 | 583 | 0.66 | 0.810 (0.779–0.842) | 1.113 | +0.796 |
| FY2023 (Apr 2023–Mar 2024) | 87,443 | 342 | 0.39 | 0.779 (0.731–0.827) | 1.009 | +0.351 |

Japanese FY runs from April 1 to March 31 (e.g., FY2020 = April 2020–March 2021) and was assigned by index (screening) date; this analysis covers the four complete fiscal years, FY2020–FY2023, which contain all 4,525 events. The crude 1-year pneumonia hospitalization rate and cohort size are shown for the full cohort; discrimination and calibration of the integer score are shown for the held-out test set.

Discrimination was broadly stable across fiscal years (integer-score AUC 0.78–0.81) despite temporal variation in the crude event rate, which peaked in FY2022. Calibration shifted across years in parallel with the changing event rate (CITL from −0.13 to +0.80), indicating that recalibration to the prevailing incidence may be required when the tool is applied in a different epidemiological period.

**Abbreviations:** AUC, area under the receiver operating characteristic curve; CI, confidence interval; CITL, calibration-in-the-large; FY, Japanese fiscal year.

## Supplementary Table 7. Competing-risk cumulative-incidence analysis of pneumonia hospitalization with non-pneumonia death as a competing event.

| Death-timing assignment | Pneumonia events | Competing deaths | AJ pneumonia CIF (%) | 1−KM (%) | AJ competing-death CIF (%) |
| --- | --- | --- | --- | --- | --- |
| Month start | 4,237 | 20,295 | 0.354 | 0.357 | 1.703 |
| Month mid (day 15) | 4,442 | 19,207 | 0.371 | 0.374 | 1.617 |
| Month end (primary) | 4,525 | 18,138 | 0.378 | 0.381 | 1.532 |

Analysis in the full otherwise-eligible risk set (n = 1,286,990) before the complete-follow-up exclusion. Death was recorded at monthly resolution; the three rows show the sensitivity of the estimate to the assigned timing of death within the month of death (start, mid [day 15], and end), with month-end as the primary analysis. The AJ estimator treats non-pneumonia death as a competing event; the complement of the Kaplan–Meier estimate (1 − KM) treats it as censoring.

In the primary (month-end) analysis, the AJ 1-year cumulative incidence of pneumonia (0.378%; 95% CI, 0.367–0.389) differed only trivially from the complement of the KM estimate treating competing death as censoring (0.381%; 0.370–0.392)—by 0.003 percentage points, well within their overlapping confidence intervals—despite non-pneumonia death being a substantially larger competing event (AJ competing-death cumulative incidence 1.53%). Competing mortality therefore leaves the marginal 1-year cumulative incidence of pneumonia essentially unchanged.

**Abbreviations:** AJ, Aalen–Johansen; CIF, cumulative incidence function; KM, Kaplan–Meier.

## Supplementary Table 8. Characteristics of included versus excluded individuals in the full otherwise-eligible cohort.

| Characteristic | Included (n = 1,098,404) | Excluded, all (n = 188,586) | Excluded: death before pneumonia (n = 18,093) | Excluded: other loss of observation (n = 170,493) | SMD (incl. vs excl.) | SMD (incl. vs death) | SMD (incl. vs other loss) |
| --- | --- | --- | --- | --- | --- | --- | --- |
| Age, years | 80.55 (4.98) | 80.02 (5.66) | 85.62 (6.33) | 79.43 (5.25) | 0.10 | -0.89 | 0.22 |
| Age 75–79 years | 50.5% | 57.4% | 20.5% | 61.4% | -0.14 | 0.66 | -0.22 |
| Age 80–84 years | 27.8% | 20.3% | 23.2% | 20.0% | 0.18 | 0.11 | 0.18 |
| Age 85–89 years | 15.7% | 14.2% | 28.0% | 12.8% | 0.04 | -0.30 | 0.08 |
| Age ≥90 years | 6.0% | 8.0% | 28.4% | 5.9% | -0.08 | -0.62 | 0.00 |
| Male sex | 40.3% | 40.6% | 52.7% | 39.3% | -0.01 | -0.25 | 0.02 |
| BMI, kg/m² | 22.84 (3.37) | 22.62 (3.54) | 21.47 (3.77) | 22.74 (3.49) | 0.06 | 0.38 | 0.03 |
| CCI score | 1.70 (1.86) | 1.96 (2.08) | 2.87 (2.48) | 1.87 (2.01) | -0.13 | -0.53 | -0.09 |
| No. of medications | 6.79 (5.47) | 6.76 (5.58) | 8.57 (6.23) | 6.56 (5.47) | 0.01 | -0.30 | 0.04 |
| QMCOO frailty-related items (12-item sum) | 2.43 (1.86) | 2.67 (2.04) | 4.22 (2.36) | 2.51 (1.93) | -0.12 | -0.84 | -0.04 |
| Poor dietary habits | 6.3% | 9.9% | 14.1% | 9.4% | -0.13 | -0.26 | -0.11 |
| Chewing difficulty | 27.3% | 28.7% | 44.0% | 27.1% | -0.03 | -0.35 | 0.00 |
| Swallowing difficulty | 20.7% | 21.3% | 29.8% | 20.4% | -0.01 | -0.21 | 0.01 |
| Weight loss | 12.0% | 14.3% | 26.1% | 13.1% | -0.07 | -0.36 | -0.03 |
| Slow walking speed | 60.2% | 60.8% | 77.3% | 59.1% | -0.01 | -0.37 | 0.02 |
| Falls | 17.8% | 20.0% | 32.7% | 18.6% | -0.06 | -0.35 | -0.02 |
| Lack of exercise habit | 36.3% | 39.9% | 59.4% | 37.8% | -0.07 | -0.48 | -0.03 |
| Memory complaints | 16.1% | 18.8% | 37.1% | 16.9% | -0.07 | -0.49 | -0.02 |
| Difficulty knowing date | 24.8% | 27.6% | 47.6% | 25.5% | -0.06 | -0.49 | -0.02 |
| Reduced going out | 10.0% | 11.8% | 30.2% | 9.8% | -0.06 | -0.52 | 0.01 |
| Limited social relations | 6.2% | 8.0% | 16.6% | 7.1% | -0.07 | -0.33 | -0.04 |
| Lack of social support | 5.3% | 6.2% | 7.2% | 6.1% | -0.04 | -0.08 | -0.04 |
| Poor self-rated health | 10.4% | 12.1% | 23.9% | 10.8% | -0.05 | -0.36 | -0.01 |
| Poor mental health | 9.8% | 11.2% | 18.9% | 10.3% | -0.04 | -0.26 | -0.02 |
| Current smoking | 5.3% | 6.9% | 6.9% | 6.9% | -0.07 | -0.07 | -0.07 |
| FY2020 | 15.1% | 1.2% | 10.4% | 0.2% | 0.53 | 0.14 | 0.58 |
| FY2021 | 68.9% | 8.7% | 72.2% | 2.0% | 1.57 | -0.07 | 1.96 |
| FY2022 | 8.1% | 86.1% | 12.9% | 93.8% | -2.50 | -0.16 | -3.34 |
| FY2023 | 8.0% | 4.0% | 4.5% | 4.0% | 0.17 | 0.14 | 0.17 |
| Integer risk score | 6.38 (2.87) | 6.60 (3.25) | 9.80 (3.39) | 6.26 (3.04) | -0.07 | -1.09 | 0.04 |
| Score ≥7 (moderate + high) | 44.3% | 46.4% | 82.5% | 42.5% | -0.04 | -0.86 | 0.04 |
| Score ≥11 (high) | 9.0% | 12.9% | 42.3% | 9.8% | -0.13 | -0.83 | -0.03 |
| Follow-up days (capped at 365) | 364.83 (5.70) | 186.83 (70.80) | 208.50 (93.26) | 184.53 (67.58) | 3.54 | 2.37 | 3.76 |

Values are mean (standard deviation) for continuous variables and % for binary variables. Excluded individuals did not satisfy the follow-up-or-event criterion of the primary cohort; “death before pneumonia” denotes death within 365 days without pneumonia hospitalization (month-end death-timing assignment), and “other loss of observation” denotes non-death disenrollment before day 365. The 18,093 deaths among excluded individuals comprise all but 45 of the 18,138 competing deaths reported in **Supplementary Table 7**; the remaining 45 occurred in included individuals whose recorded death month ended exactly at day 365 and who therefore satisfied the complete-follow-up criterion. The integer risk score was computed for all individuals from the final scorecard. SMDs are (mean₁ − mean₂)/√[(v₁+v₂)/2]; no significance tests were performed.

**Abbreviations:** BMI, body mass index; CCI, Charlson Comorbidity Index; FY, fiscal year; QMCOO, Questionnaire for Medical Checkup of Old-Old; SMD, standardized mean difference.

## Supplementary Table 9. Full-eligible-cohort sensitivity evaluation of the final models with death as a competing event and IPCW.

| Metric | Super Learner | Integer risk scorecard |
| --- | --- | --- |
| Test-set AUC (complete follow-up) | 0.823 | 0.786 |
| Full-cohort IPCW-weighted AUC (95% CI) | 0.818 (0.806 to 0.829) | 0.782 (0.768 to 0.794) |
| Change in AUC vs test set (95% CI) | -0.005 (-0.009 to -0.002) | -0.003 (-0.008 to 0.000) |
| Calibration slope (95% CI) | 1.027 (0.986 to 1.067) | 0.971 (0.922 to 1.014) |
| CITL (95% CI) | -0.098 (-0.157 to -0.044) | -0.080 (-0.139 to -0.024) |
| IPCW-weighted Brier score | 0.00383 | 0.00384 |
| Mean predicted risk, % | 0.434 | 0.427 |
| Observed risk (IPCW-weighted), % | 0.388 | 0.388 |
| Observed risk (AJ), % | 0.379 | 0.379 |
| E/O ratio (95% CI) | 1.12 (1.06 to 1.19) | 1.10 (1.04 to 1.17) |
| Age ≥90 years (n = 24,318): IPCW-weighted AUC | 0.673 | 0.654 |
| Age ≥90 years: calibration slope | 0.683 | 0.590 |
| Age ≥90 years: CITL | −0.107 | +0.312 |
| Age ≥90 years: E/O ratio | 1.11 | 0.74 |

Evaluation set n = 386,099 (held-out test set n = 329,524 plus a stratified random 30% sample [n = 56,575] of the 188,586 excluded individuals); no individual used for model fitting contributed. Death before pneumonia was treated as a known non-event; non-death loss of observation was handled by IPCW (censoring Cox model conditional on all 20 candidate predictors, fiscal year, and the final scorecard’s linear predictor), with weights truncated at the 1st/99th percentiles (untruncated weights gave nearly identical results; weight diagnostics: median 1.00, 99th percentile 3.40, maximum 14.5, effective sample size 279,505 of 334,952 outcome-ascertainable individuals). Confidence intervals are bootstrap percentile intervals (B = 500) re-estimating the censoring model in each replicate, conditional on the final models. CITL >0 indicates observed risk exceeding predicted risk on the logit scale. Results were qualitatively similar across the three within-month death-timing assignments. In the evaluation set, the AJ 1-year incidence was 0.11%, 0.45%, and 1.64% in the low-, moderate-, and high-risk score groups (IPCW-weighted: 0.12%, 0.47%, and 1.72%).

**Abbreviations:** IPCW, inverse probability of censoring weighting; AUC, area under the receiver operating characteristic curve; CI, confidence interval; CITL, calibration-in-the-large; AJ, Aalen–Johansen; E/O, ratio of mean predicted to observed risk.

## Supplementary Table 10. Stability of the 17 scorecard logistic regression coefficients when refitted with IPCW weights in the full-cohort development analogue.

| Term | Original coefficient (complete follow-up) | Full-cohort IPCW coefficient | Bootstrap 95% CI | Change, % | Direction unchanged |
| --- | --- | --- | --- | --- | --- |
| Intercept | -11.299 | -11.201 | -11.940 to -10.572 | – | – |
| Male sex | 1.183 | 1.154 | 1.070 to 1.224 | -2.5 | Yes |
| BMI | -0.126 | -0.116 | -0.130 to -0.104 | +7.8 | Yes |
| Age | 0.081 | 0.077 | 0.071 to 0.084 | -4.6 | Yes |
| CCI score | 0.113 | 0.098 | 0.082 to 0.113 | -12.7 | Yes |
| Lack of exercise habit | 0.456 | 0.457 | 0.376 to 0.534 | +0.2 | Yes |
| No. of medications | 0.032 | 0.032 | 0.026 to 0.038 | -1.6 | Yes |
| Memory complaints | 0.370 | 0.334 | 0.244 to 0.425 | -9.8 | Yes |
| Difficulty knowing date | 0.272 | 0.251 | 0.163 to 0.326 | -7.5 | Yes |
| Reduced going out | 0.376 | 0.308 | 0.205 to 0.393 | -18.1 | Yes |
| Falls | 0.248 | 0.237 | 0.166 to 0.320 | -4.3 | Yes |
| Chewing difficulty | 0.202 | 0.195 | 0.118 to 0.280 | -3.2 | Yes |
| Slow walking speed | 0.155 | 0.203 | 0.113 to 0.292 | +31.2 | Yes |
| Swallowing difficulty | 0.158 | 0.157 | 0.084 to 0.236 | -0.9 | Yes |
| Weight loss | 0.155 | 0.098 | 0.006 to 0.198 | -36.3 | Yes |
| Self-rated health | 0.135 | 0.118 | 0.010 to 0.223 | -12.2 | Yes |
| Limited social relations | 0.109 | 0.091 | -0.014 to 0.186 | -16.4 | Yes |
| Dietary habits | 0.121 | 0.132 | 0.030 to 0.252 | +9.4 | Yes |

The same 17 features and coding as the original scorecard logistic model were refitted with IPCW weights in the full-cohort development analogue (n = 900,891: the development set plus the 70% of excluded individuals not sampled into the evaluation set), treating death before pneumonia as a non-event and weighting non-death loss by the inverse probability of remaining under observation. Bootstrap confidence intervals (B = 200) re-estimated the censoring model in each replicate. All 17 coefficient directions were preserved (median absolute change 7.8%). Sixteen of 17 original coefficients fell within the bootstrap 95% CI; the exception was the CCI coefficient, whose original value (0.11284) lay marginally above the upper bootstrap bound (0.11256) — at the displayed precision both round to 0.113. **Abbreviations:** BMI, body mass index; CCI, Charlson Comorbidity Index; CI, confidence interval; IPCW, inverse probability of censoring weighting.

## Supplementary Table 11. Decile-level calibration of the final models in the full-eligible-cohort evaluation.

| Model | Bin | n | Mean predicted risk, % | Observed (AJ), % | Observed (IPCW-weighted), % |
| --- | --- | --- | --- | --- | --- |
| Super Learner | 1 | 38,610 | 0.056 | 0.023 | 0.028 |
|  | 2 | 38,610 | 0.082 | 0.061 | 0.058 |
|  | 3 | 38,610 | 0.113 | 0.072 | 0.070 |
|  | 4 | 38,610 | 0.147 | 0.054 | 0.050 |
|  | 5 | 38,610 | 0.189 | 0.131 | 0.140 |
|  | 6 | 38,609 | 0.244 | 0.207 | 0.236 |
|  | 7 | 38,610 | 0.322 | 0.264 | 0.282 |
|  | 8 | 38,610 | 0.449 | 0.419 | 0.417 |
|  | 9 | 38,610 | 0.706 | 0.695 | 0.733 |
|  | 10 | 38,610 | 2.037 | 1.856 | 1.925 |
| Integer risk scorecard | 1 | 62,028 | 0.070 | 0.041 | 0.042 |
|  | 2 | 47,719 | 0.114 | 0.085 | 0.085 |
|  | 3 | 52,485 | 0.158 | 0.141 | 0.151 |
|  | 4 | 51,498 | 0.220 | 0.190 | 0.198 |
|  | 5 | 46,265 | 0.305 | 0.248 | 0.258 |
|  | 6 | 38,054 | 0.423 | 0.445 | 0.452 |
|  | 7 | 29,623 | 0.586 | 0.584 | 0.621 |
|  | 8 | 21,594 | 0.811 | 0.738 | 0.756 |
|  | 9 | 36,833 | 1.914 | 1.645 | 1.725 |

Bins are deciles of predicted risk in the evaluation set (n = 386,099); for the integer scorecard, ties in the discrete score reduce the number of distinct bins to nine. Observed risks are the within-bin AJ 1-year cumulative incidence (death as a competing event) and the within-bin IPCW-weighted event rate; their agreement indicates that the calibration assessment is not sensitive to the handling of censoring.

**Abbreviations:** AJ, Aalen–Johansen; AUC, area under the receiver operating characteristic curve; IPCW, inverse probability of censoring weighting.

## Supplementary Table 12. Complete point-assignment specification of the 17-feature integer risk scorecard.

| Feature | Category / range | Points |
| --- | --- | --- |
| Male sex | No | 0 |
|  | Yes | 1 |
| BMI, kg/m² | ≤20.5 | 2 |
|  | 20.6–24.9 | 1 |
|  | >24.9 | 0 |
| Age, years | ≤76 | 0 |
|  | 77–84 | 1 |
|  | ≥85 | 2 |
| CCI score | ≤1 | 0 |
|  | 2–3 | 1 |
|  | ≥4 | 2 |
| Lack of exercise habit | No | 0 |
|  | Yes | 1 |
| No. of medications | ≤3 | 0 |
|  | 4–9 | 1 |
|  | ≥10 | 2 |
| Memory complaints | No | 0 |
|  | Yes | 1 |
| Difficulty knowing date | No | 0 |
|  | Yes | 1 |
| Reduced going out | No | 0 |
|  | Yes | 1 |
| Falls | No | 0 |
|  | Yes | 1 |
| Chewing difficulty | No | 0 |
|  | Yes | 1 |
| Slow walking speed | No | 0 |
|  | Yes | 1 |
| Swallowing difficulty | No | 0 |
|  | Yes | 1 |
| Weight loss | No | 0 |
|  | Yes | 1 |
| Self-rated health | Good | 0 |
|  | Poor | 1 |
| Limited social relations | No | 0 |
|  | Yes | 1 |
| Dietary habit (eat less) | No | 0 |
|  | Yes | 1 |

Binary QMCOO items are scored 0 (no) or 1 (yes). Continuous features use quartile cutpoints fixed on the development set; the lowest and highest categories are open-ended for application, and age was capped at 100 years. Adjacent quartiles that received equal points are shown as a single band. BMI is recorded to one decimal place, so the BMI categories are contiguous. In the development and full otherwise-eligible cohorts, no value fell outside the development ranges (BMI 10–50 kg/m²; age 75–100 years; CCI 0–18; medications 0–91). In the underlying logistic model, continuous features enter untransformed (age in years, BMI in kg/m², CCI score, and medication count); the intercept is −11.299, with coefficients in Supplementary Table 3. The total score is the sum over all 17 features (range 0–21). Risk groups: low 0–6, moderate 7–10, high ≥11. The underlying 17-feature logistic coefficients are given in **Supplementary Table 3**; the raw logistic probability is calibrated as p = expit (−5.747 + 36.963 × p_raw), a logistic recalibration applied on the probability scale, fitted on the development set.

**Abbreviations:** BMI, body mass index; CCI, Charlson Comorbidity Index; QMCOO, Questionnaire for Medical Checkup of Old-Old.

## Supplementary Table 13. Lookup table mapping each integer score (0–21) to the calibrated 1-year predicted probability of pneumonia hospitalization.

| Score | Predicted probability, % | Risk group | Development n | Development events |
| --- | --- | --- | --- | --- |
| 0 | 0.031 | Low | 1,319 | 0 |
| 1 | 0.043 | Low | 11,870 | 7 |
| 2 | 0.059 | Low | 37,903 | 18 |
| 3 | 0.082 | Low | 71,179 | 43 |
| 4 | 0.114 | Low | 95,564 | 99 |
| 5 | 0.158 | Low | 105,913 | 155 |
| 6 | 0.220 | Low | 104,740 | 220 |
| 7 | 0.305 | Moderate | 92,701 | 300 |
| 8 | 0.423 | Moderate | 77,518 | 325 |
| 9 | 0.586 | Moderate | 58,813 | 371 |
| 10 | 0.811 | Moderate | 42,534 | 380 |
| 11 | 1.123 | High | 28,692 | 313 |
| 12 | 1.552 | High | 18,326 | 320 |
| 13 | 2.142 | High | 10,800 | 243 |
| 14 | 2.950 | High | 5,911 | 149 |
| 15 | 4.049 | High | 3,028 | 107 |
| 16 | 5.535 | High | 1,326 | 58 |
| 17 | 7.523 | High | 518 | 32 |
| 18 | 10.148 | High | 165 | 14 |
| 19 | 13.555 | High | 41 | 10 |
| 20 | 17.879 | High | 17 | 1 |
| 21 | 23.211 | High | 2 | 1 |

Predicted probabilities are given by p(S) = expit(−8.087531 + 0.328148 × S), a logistic calibration of the integer total score S fitted on the development set only (six-decimal coefficients, sufficient to reproduce every tabulated value). On the held-out test set this mapping showed calibration slope 1.017, CITL −0.001, and Brier score 0.0041. Development n and events are shown for reference.

**Abbreviations:** CITL, calibration-in-the-large.

## Supplementary Figure 1. ROC curves of (a) the Super Learner ensemble and (b) the 17-feature integer risk scorecard in the test set.

The Super Learner achieved an AUC of 0.823 (95% confidence interval, 0.812–0.834) and the integer risk scorecard an AUC of 0.786 (0.774–0.798).

**Abbreviations:** ROC, receiver operating characteristic; AUC, area under the curve.

## Supplementary Figure 2. Calibration plots of (a) the Super Learner ensemble and (b) the 17-feature integer risk scorecard in the test set.

Predicted probabilities (x-axis) are plotted against observed event rates (y-axis) across 20 bins of predicted risk. The dashed diagonal line represents perfect calibration. The Super Learner had a calibration slope of 1.101 (CITL −0.002) and the integer score a calibration slope of 1.017 (CITL −0.001).

**Abbreviation:** CITL, calibration-in-the-large.

## Supplementary Figure 3. Incremental feature selection by development-internal 10-fold cross-validation for the three ranking methods (LASSO, ridge, and ANOVA).

The x-axis is the number of selected features and the y-axis is the development 10-fold cross-validated area under the curve. The cross-validated AUC plateaus beyond roughly ten features; ridge-based selection with 17 features (circled) gave the highest cross-validated AUC and was chosen for the final scorecard.

**Abbreviations:** AUC, area under the curve.

## Supplementary Figure 4. Decision curve analysis comparing the net benefit of the 17-feature integer risk scorecard against treat-all and treat-none strategies across a range of threshold probabilities in the test set.

The solid line represents the risk scorecard, the dashed line represents the treat-all strategy, and the dotted line represents the treat-none strategy. Net benefit is expressed per 1,000 individuals.

## Supplementary Figure 5. Competing-risk cumulative incidence of pneumonia hospitalization and non-pneumonia (competing) death over 1 year, with 95% confidence intervals.

Aalen–Johansen cumulative incidence functions in the full otherwise-eligible risk set (n = 1,286,990). Shaded bands are 95% confidence intervals. Non-pneumonia death (competing, 1.53%) is a larger competing event than pneumonia hospitalization (0.38%), yet the pneumonia cumulative incidence (0.378%; 95% CI, 0.367–0.389) is nearly identical to the censoring-based estimate (0.381%), confirming that competing mortality does not materially affect the marginal 1-year cumulative incidence of pneumonia.
